# Supplementary material for: FGF-23 as a Biomarker for Carotid Plaque Vulnerability: A Systematic Review
Source: Med Sci (Basel). 2025 Mar 10;13(1):27. doi: 10.3390/medsci13010027 (PMC11943977; doi:10.3390/medsci13010027)
Supplement: Supplementary file 1 [file medsci-13-00027-s001.zip › medsci-3501305-supplementary.pdf]

Supplementary Tables

Table 1S. Search query – key words

**Bibliographic source**	**Search term**	**Nº of reports**<br>1/10/2024
MEDLINE	("Endarterectomy" [MeSH Terms] OR "Endarterectomy, Carotid" [MeSH Terms] OR "Carotid Endarterectomies" [MeSH Terms] OR "Carotid Endarterectomy" [All Fields])<br>AND<br>("Fibroblast Growth Factor-23" [MeSH Terms] OR "FGF23" [All Fields] OR "Fibroblast Growth Factor 23" [All Fields])	5
Web of Science	("Endarterectomy" OR "Endarterectomy, Carotid" OR "Carotid Endarterectomies" OR "Carotid Endarterectomy")<br>AND<br>("Fibroblast Growth Factor-23" OR "FGF23" OR "Fibroblast Growth Factor 23")	4
SCOPUS	("Endarterectomy" OR "Endarterectomy, Carotid" OR "Carotid Endarterectomies" OR "Carotid Endarterectomy")<br>AND<br>("Fibroblast Growth Factor-23" OR "FGF23" OR "Fibroblast Growth Factor 23")	11

Table 2S. Covariates used in the adjusted models

Author	Covariables used in adjusted models
Biscetti, F. et al	Sex, Age, Smoking, Hypertension, Hypercholesterolemia, Triglycerides, LDL-C, HsCRP and IL-6 (Model 1) plus FGF23 and OPG (Model 2)
Del Porto, F. et al	NA
Zamani, M. et al	eGFR, FGF23

LDL-C – Low-Density Lipoprotein Cholesterol; HsCRP – High-Sensitivity C-Reactive Protein; IL – Interleukin; FGF23 – Fibroblast Growth Factor-23; OPG – Osteoprotegerin; eGFR – Glomerular Filtration Rate; NA – Not Available
